# Autonomous localized path planning algorithm for UAVs based on TD3 strategy

**DOI:** 10.1038/s41598-024-51349-4

**Published:** 2024-01-08

**Authors:** Zhao Feiyu, Li Dayan, Wang Zhengxu, Mao Jianlin, Wang Niya

**Affiliations:** Faculty of Information Engineering and Automation, Kunming University of Science and Technology, Kunming, China

**Keywords:** Engineering, Electrical and electronic engineering

## Abstract

Unmanned Aerial Vehicles are useful tools for many applications. However, autonomous path planning for Unmanned Aerial Vehicles in unfamiliar environments is a challenging problem when facing a series of problems such as poor consistency, high influence by the native controller of the Unmanned Aerial Vehicles. In this paper, we investigate reinforcement learning-based autonomous local path planning methods for Unmanned Aerial Vehicles with high autonomous decision-making capability and locally high portability. We propose an autonomous local path planning algorithm based on the TD3 strategy to solve the problem of local obstacle avoidance and path planning in unfamiliar environments using autonomous decision-making of Unmanned Aerial Vehicles. The simulation results on Gazebo show that our method can effectively realize the autonomous local path planning task for Unmanned Aerial Vehicles, the success rate of path planning with our method can reach 93% under the interference of no obstacles, and 92% in the environment with obstacles. Finally, our method can be used for autonomous path planning of Unmanned Aerial Vehicles in unfamiliar environments.

## Introduction

Currently, the methods to achieve Unmanned Aerial Vehicle (UAV) trajectory control are quite mature with the help of GPS to achieve its own positioning^1,2^ or real-time optimization of its position with the help of SLAM^3–5^ used geographic information systems (GIS) as the DRL training environment to overcome the inconsistency between the training environment and the test environment, which achieved UAV deciding a track by itself in a complex geometrical environment. Cimurs et al.^6^ presented an autonomous navigation system for goal-driven exploration of unknown environments for unmanned vehicles through deep reinforcement learning. Zhou et al.^7,8^propose an efficient quadrotor autonomous path planning method based on the underlying control to realize the fast planning work of UAVs in unfamiliar environments. However, most of these algorithms rely on control algorithms as well as manual algorithmic decision-making to enable the UAV to accomplish obstacle avoidance, and the UAV does not yet have an autonomous decision-making process and may experience poor obstacle avoidance due to controller changes. Comparison with other relevant recent work^5^, training with GIS, so that the portability of its algorithm is low, while in the deployment of the real machine is going to have greater difficulties, our algorithm is trained in gazebo, in the deployment of the real machine will be more convenient; Cimurs et al.^6^ in the only training in two-dimensional space, and the lack of the camera’s auxiliary localization and target search function, this paper training will always be the camera output data is also as important as the environmental information to conduct training; and algorithms such as^7,8^, more from the UAV’s native controller to start, in the native control to realize the UAV’s autonomous local path planning work, while we do not change the native controller, reinforcement learning agent only as a decision module, greatly improving the portability of the algorithm.

To plan a path, a robot needs to find the target, assess the environment, and determine the best course of action based on its state. This becomes challenging for UAVs when flying at different altitudes without depth information. Monocular cameras can also distort images, making it difficult to gauge real-world scale. In the meantime, UAVs use speed and acceleration control methods with fixed-point control to reach the target directly above it. However, when distance information is missing, depth information is obtained from consecutive photos, and the relative position between the UAV and the target is solved. Converting this information allows high-level controllers to control the UAV, but adjustments are needed to account for different cameras and heights.

Aiming at these problems, a deep reinforcement learning (DRL) autonomous local path planning pipeline was proposed in this paper, which realizes automatic path planning and successfully reaches the target point by detecting the relative coordinates and relative positions between the target point and the local coordinate system of the UAV through a monocular camera and calculating the relative positions at different heights. The RL Intelligent Body (RL Agent) fuses the image information output from the target detection module with the altitude information provided by the sensors and inputs it to the RL Agent and finally outputs advanced control information to the controller of the cooperative operating system so that the UAV can efficiently and accurately complete the local path planning task. The algorithms in this paper are useful when adapting to multiple types of UAVs (with different underlying controllers) or when the UAV is equipped with only a monocular camera and a rudimentary LIDAR or when the UAV’s onboard computing device is not GPU-accelerated, or when all three of the above characteristics are present for the localized path planning work. The contributions of this paper are as follows:

**Figure 1:**
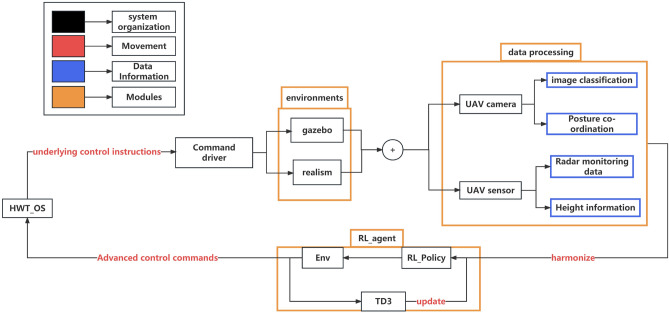
Block diagram of RL agent system.

- Proposing an RL strategy algorithm for the system for autonomous local path planning. With the help of this algorithm, it is possible to realize a capability that has a high degree of portability and allows the UAV to make autonomous decisions and realize path planning without changing the native controller of the UAV;
- Includes a robot advance controller system for the Unmanned Aerial Vehicles‘ flight control. With this system, the underlying flight controllers of various UAVs can be better adapted to better improve the portability of the algorithms in this paper;
- Proposing a Gazebo simulation environment to test the algorithms in this paper, which includes the training environment and testing environment to be suitable for the RL agent training.

## Related works

With the development of deep reinforcement learning (DRL), there is an increasing number of various works on reinforcement learning applied to robotics^5,6,9^. Reinforcement learning has been successful in the field of FPV racing sports as well^10,11^. And for or trajectory optimization, a series of effective strategies have also been proposed in recent years to solve the trajectory optimization problems^12–17^. For quadcopters, there are also some excellent algorithms that have been proposed in the recent past to enable autonomous UAV deer in unfamiliar environments^7,8^, which can also show good results in cluster operation^18^. With the continuous development and advancement of real-time target detection^19,20^, cameras^21^, lidar, and deep reinforcement learning algorithms^22–24^, the cost of deploying reinforcement learning to robots is gradually decreasing and its feasibility is gradually increasing, and a lot of work on integrating DRL with robotics has emerged. In Ref.^9^, the target point is defined as a semantic network result and the semantic image is fused with the depth image. Finally, the results are fed into the RL strategy for decision-making, which ultimately results in a fast and accurate emergency landing of the UAV. In^6^, the authors used random coordinates as the form of the target point for the UAV. After the unmanned vehicle acquires the target point, according to the distance information returned by the LiDAR, the information is fused with the car’s own sensor data, and then input into the RL Agent to output the decision, realizing the autonomous navigation function of the unmanned vehicle. Although these works have yielded good results, most of them require depth information to solve for the relative positions of the target points or assume that the relative positions of the target points have been obtained by other methods.

In this paper, we present an autonomous local path planning method that addresses the challenge of UAV path planning with only image data. Our approach is based on the deep reinforcement learning (DRL) algorithm and solves the issue of inaccurate relative positioning when the UAVs are at different altitudes and there is no target depth information available. The proposed method enables UAVs to efficiently and accurately reach their targets and complete localized path-planning tasks.

The remainder of this paper is organized as follows. The research direction of this thesis was introduced in Introduction. The work related to reinforcement learning and path planning was described in “Related works”. The issues encountered and the system model is described in “Problem description and system modelling”. The path planning algorithm of this paper is presented in “Path planning algorithm”. The relevant data and results of the experiments are given in “Result analysis”. Finally, The conclusions and future works are drawn in “Conclusion and future work”.

## Problem description and system modelling

This paper is dedicated to solving the problem of designing decision-making models to help UAVs realize local path planning work in unfamiliar environments based on environmental information without changing the native controller of the UAV. This paper formulates it as a deep reinforcement learning problem, in which the RL Agent extracts preprocessed environmental information and sensor information for training, so that the RL Agent can utilize the environmental information to enable the UAV to accurately find the relative position information and make the path planning in the absence of GPS signals as well as target depth information.

In view of the introduction of yolov7^25^, this paper adapts its interface to the RL Agent, which greatly reduces the training resources, and can be deployed on the cpu platform to complete the training phase. To train the model, this paper uses a realistic physical model provided by px4^26^. The advanced control commands are outputted to the HWT_OS system, which can be quickly deployed to the real UAV environment after fine-tuning the parameters on the basis of the simulation training and when the camera internal parameters are kept consistent.

### RL agent system model

As shown in Fig. 1, the environment information is transmitted to the data preprocessing module from the simulation environment or the real machine. In order to improve the training speed, the work in this paper does not hand over the image information, sensor information, and lidar information to the RL Agent for processing, but the data are handed over to the data preprocessing module for preprocessing. The data preprocessing and fusion module preprocesses and fuses the image information, sensor information, and lidar information as the environment information to be processed by the RL Agent, which makes corresponding planning according to the current environment information and outputs the actions to the HWT_OS system, which will decode the control information of the UAV into the control commands of the mavros and finally output the bottom control information to the UAV. The modularized approach used in this paper with HWT_OS can be used to port the algorithm to other robotic devices more quickly, and with the advantage of HWT_OS, the development of multi-machine cooperative work can be carried out at a later stage.

**Figure 2:**
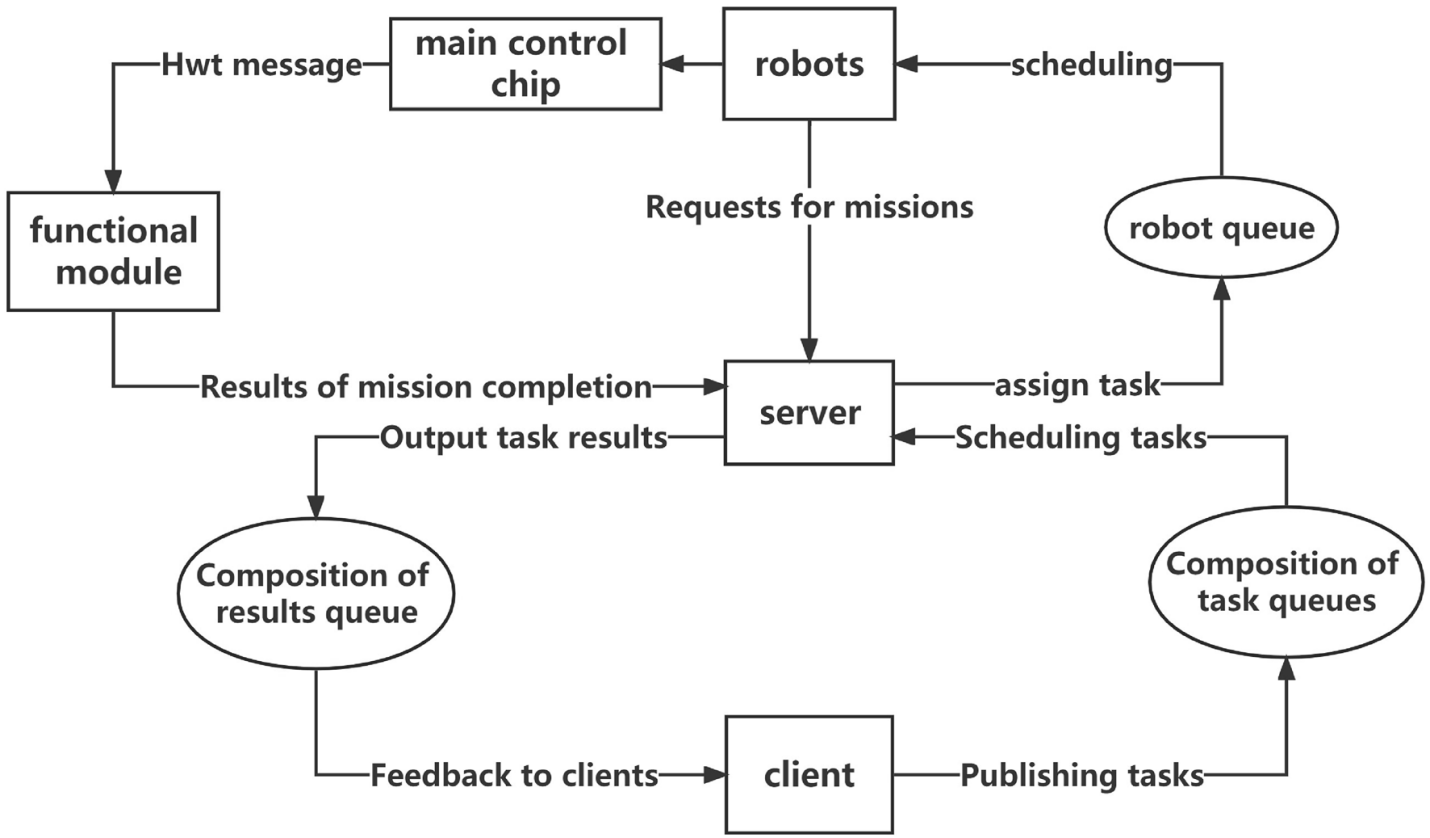
HWT_OS flowchart.

### HWT_OS system

HWT_OS is a set of UAV-implemented cluster control system established at the beginning of this work. As shown in Fig. 2, the unified information data structure as well as the communication protocols allow the newly added robots to be more quickly integrated into the existing cluster control system. The work in this paper uses only parts of its advanced controller for better control of UAVs and uses its env modules to provide interfaces to adapt RL algorithms to his calls.

The UAV advanced controller module of HWT_OS integrates the interfaces of PX4-MAVLINK-ROS-MAVROS and provides additional ENU, body, etc. for controlling the speed, position, and acceleration of the UAV in different combinations under multiple coordinate systems, and provides interfaces on the ROS side to enable external operations and their convenient use of commands to control the UAV in multiple coordinate systems to different trajectories movement in multiple coordinate systems. The env modules are specially designed to provide an interface for gazebo simulation, integrating the advanced controller interface of HWT_OS, which can be uniformly delivered to gazebo for manipulating the robot’s initialization, movement, and obstacle changes.

## Path planning algorithm

**Figure 3:**
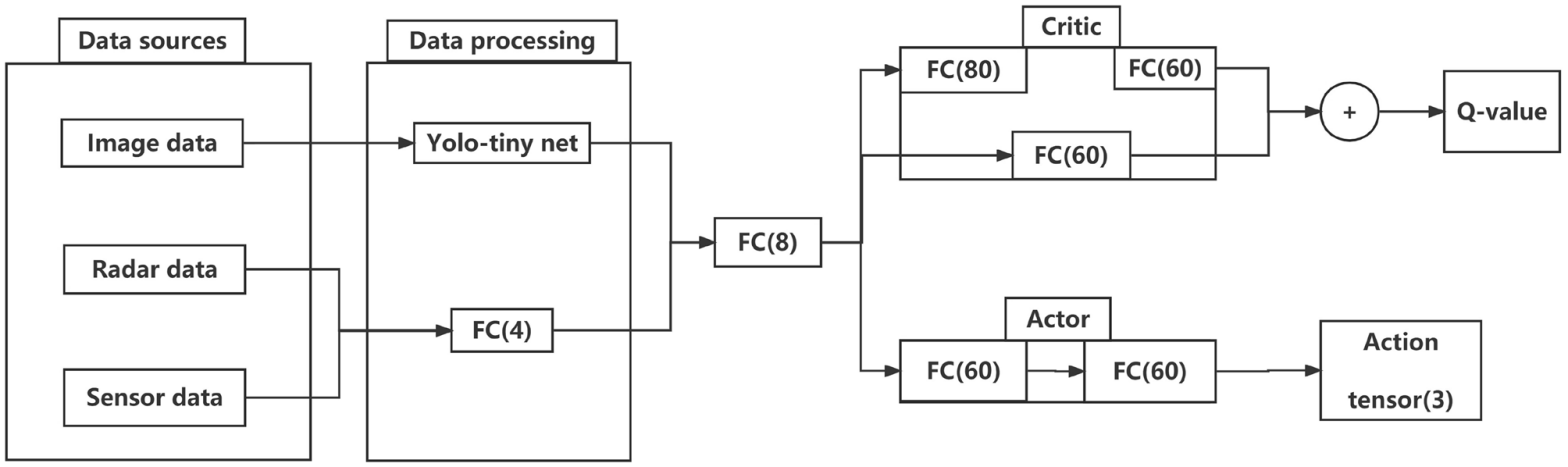
Policy framework.

The RL Agent uses the Actor-Critic model to map the preprocessed environmental information to the UAV actions, and its architecture is shown in Fig. 3. In it, each action includes a high-level control instruction (control mode (velocity or displacement), control coordinate system (ENU system or body system), value (velocity value or displacement value)), and each set of values can be selected among the specified thresholds. In the following, we describe in more detail the individual modules of the algorithm.

### Algorithmic architecture

Actor and Critic networks share the environment information provided by the data preprocessing and fusion module. The Actor network consists of three fully connected (FC) layers, the last part of which outputs a set of 4-dimensional actions, and in order to make the output action intervals in the simultaneous existence of positive and negative value selection, the activation function of the last FC layer selects the tanh function; the Critic network consists of two networks composed of four FC layers respectively, and both networks output one-dimensional Q-value values, and the RL Agent adopts the policy of TD3^24^ to update parameters. The Critic network consists of two networks consisting of 4 FC layers, both of which output one-dimensional Q-value values, and the RL Agent adopts the strategy of TD3^24^ to update the parameters, one of which is used to send the Q-value to the Actor network to update the parameters of the Actor network, and the other one is used to update the parameters of the Critic itself in batches during the iteration process. At the same time, in order to improve the training speed and accelerate the training speed of the CPU side, this paper does not add the convolutional layer in the RL training network, and all the preprocessing process of the environmental information is handed over to the data fusion layer and reduces the redundancy degree of the data in the data fusion layer in order to improve the training speed.

### Data preprocessing and fusion

The data preprocessing and fusion module provides appropriate state information to the RL Agent to better adapt the reinforcement learning algorithm, which serves to accelerate the training while also better preparing for future expansion and porting to other robotics platforms.

This module integrates yolov7 outputs data, lidar data of UAV, and sensor data of UAV. The three are processed and fused to get the STATE information and provided to the RL Agent.

For reinforcement learning algorithms for simulation applications, each time step takes longer to complete compared to other training algorithms. Directly outputting redundant information such as image and lidar information to the RL Agent can significantly increase the time required for each time step and also prolong the fitting time for training. In the fifth part of this paper, we will compare the experimental data of outputting the redundant information and the fused information.

In order to solve this problem, this paper uses the yolov7-tiny model, and single-step detection as the method, cancels the real-time monitoring to improve the training speed, provides an interface, each time the data fusion is performed, calls the interface to get the yolov7-tiny output image as well as the tensor vector. Only the part of the vector containing coordinate information and category information is intercepted, and a set of 5-dimensional tensor-img vectors is obtained after fusion, and the image information is used to visualize the observation and comparison.

In this paper, the same data fusion method is used for the sensor information, and only the current altitude information is extracted for its own basic information, yaw angle information and lidar data. Lidar information has the highest redundancy among the sensor information, considering that the RL Agent adopts single-step planning, this paper, on the basis of eliminating the inf and other useless data, additionally eliminates all the values other than the shortest distance direction, and only retains the shortest distance and the shortest distance direction, so as to reduce the redundancy of the information, and to improve the convergence speed.

### Rewards

There are Markovian properties for single-step planning of local path planning, so in this paper, each step of path planning is considered as a Markovian decision-making process.The training of the RL Agent is based on the data and rewards collected at each decision. At each decision, the total reward is composed of different terms as follows:

1 $$\begin{aligned} R= & {} R_t+w_n\cdot R_n+R_s+w_C\cdot Rc, \end{aligned}$$

where $R_t$ is the final reward, $R_n$ is the step reward, $R_s$ is the single step reward, $R_c$ is the collision reward, and $W_n$, $W_c$ are the weights of the step reward as well as the collision reward, respectively.

The final reward $R_t$ refers to a fixed 100-point reward given to the UAV when it reaches the point directly above the target point.

2 $$\begin{aligned} R_{t}= & {} \left\{ \begin{array}{l} 100, \text{ if } \text{ goal } \\ 0, \text{ otherwise } \end{array}\right. \end{aligned}$$

The aim is to encourage the drone to reach directly above the goal point without collision.

The step reward $R_n$ refers to the ratio between the maximum number of steps the drone can move and the number of steps it is currently moving under the current curtain.

3 $$\begin{aligned} R_n= & {} -count(step)/maxstep, \end{aligned}$$

This reward’s encourages the drone to travel faster to the target point, while improving the convergence speed of the algorithm.

The single-step reward, $R_s$, is the reward that changes most frequently at each time step, which calculates whether the planning of this step keeps the drone close to the target point or not.

4 $$\begin{aligned} R_s= & {} -\left\{ \begin{array}{ll} (w_P \sqrt{(x_P-x_{Pc})^2+(y_P-y_{Pc} )^2 )} + w_E \sqrt{(x_E-x_{Ec} )^2+(y_E-y_{Ec} )^2)}, &{}\quad \text {if not goal}\\ -10-\ w_E\ \sqrt{(x_E-x_{Ec})^2+(y_E-y_{Ec})^2},&{}\quad \text {if the target is lost} \\ 0,&{}\quad \text {if goal} \end{array}\right. \end{aligned}$$

$X_P$, $Y_P$ are the coordinate values of the target center in the image coordinate system, $X_{Pc}$, $Y_{Pc}$ are the coordinate values of the center point of the image, $W_p$ is the weights of the pixel error, $X_E$, $Y_E$ are the coordinate values of the target center at the target point in the ENU coordinate system, $X_{Ec}$, $Y_{Ec}$ are the coordinate values of the current position of the UAV in the ENU coordinate system, and We is the weights of the ENU error. Adding the correction under the ENU system in the but-step reward can increase the convergence speed and reduce the training cost. This reward is mostly negative reward plays the role of encouraging UAV exploration.

The collision reward $R_c$ refers to the UAV’s penalty for the current curtain in the event of a collision.

5 $$\begin{aligned} R_c= & {} \left\{ \begin{array}{ll} 1,&{}\quad \text {if collision} \\ 0, &{}\quad \text {otherwise} \end{array}\right. \end{aligned}$$

### Network update strategy

Reinforcement learning discusses the problem of how an agent can maximize the rewards it can obtain inside a complex and uncertain environment. By sensing the *STATE* of the environment it is in and the rewards *REWARD* obtained from the action chosen at that moment, the parameter network is updated to obtain a better *REWARD*.

**Figure 4:**
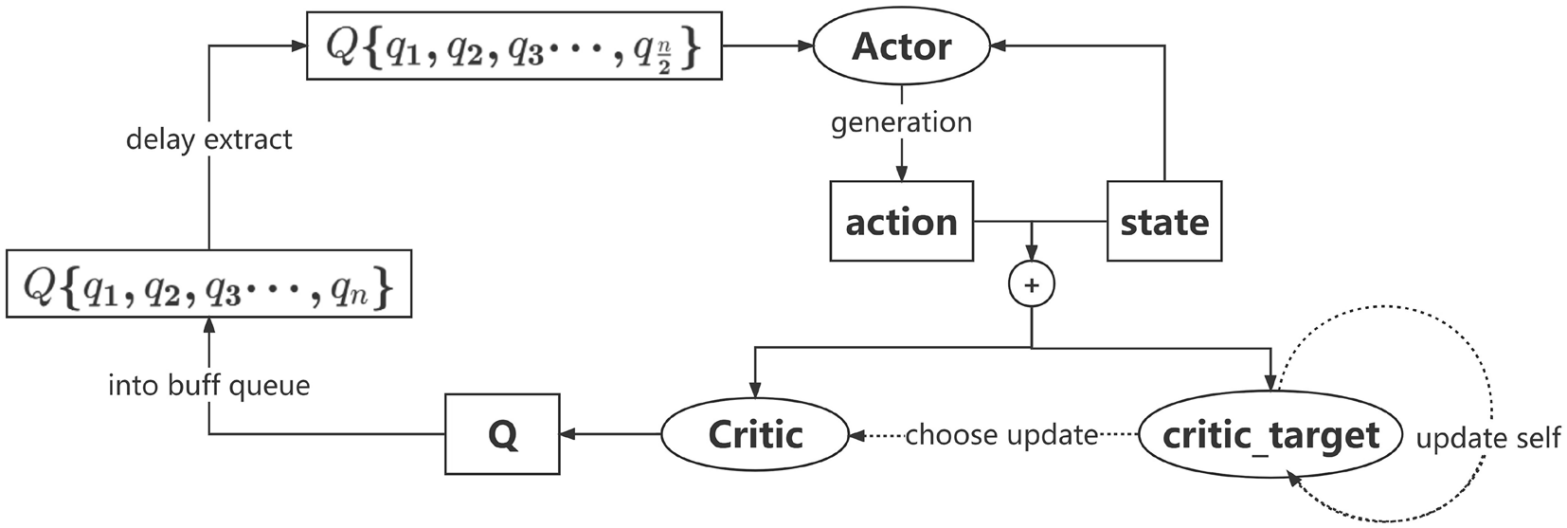
TD3 algorithm AC framework.

The TD3 algorithm adopted in this paper draws on the training idea of Double DQN^22^, utilizes the Actor network and Critic network in the AC algorithm, and the basic framework of the algorithm is shown in Fig. 4, which is combined with the Bellman equation to realize the autonomous local path planning of the UAV under continuous motion.

In Fig. 4, in TD3 algorithm, Actor network Actor generates the strategy, and Critic network Critic evaluates and improves the current strategy by estimating the dominance function An. Both are optimized according to the strategy gradient, and the updating formulas for the parameters $\theta (a)$ of Actor network, and the parameters $\varphi (c)$ of Critic network are as follows:

6 $$\begin{aligned} \nabla _\varphi J(\varphi )= & {} E_{s\sim p_\pi }[\nabla _aQ^\pi (s,a)|_{a = \pi (s)}\nabla _\varphi \pi _\varphi (s)] \end{aligned}$$

The gain function in the above equation values the Actor strategy and calculates the gain expectation $R_0$ obtained by the agent with state transfer and action selection after adopting this strategy.

The maximized gain function is as follows:

7 $$\begin{aligned} J(\varphi )= & {} E_{s_i \sim p_\pi ,a_i \sim \pi }(R_0) \end{aligned}$$

Critic calculates the earnings valuation function as follows:

8 $$\begin{aligned} Q^\pi (s,a)= & {} E_{s\sim p_\pi ,a\sim \pi }[R_t|s,a] \end{aligned}$$

The continuous gain equation depicts the relationship between the gain of a pair (s, a) at the current moment and the gain of a pair (s’, a’) at the next moment:

9 $$\begin{aligned} Q^\pi (s,a)= & {} r+\gamma E_{s',a'}[Q^\pi (s',a')],a'\sim \pi (s') \end{aligned}$$

### Ethics statement

Participants’ informed consent: We explained the purpose, process, risks, and benefits of the study to all individuals participating in the study either orally or in writing, and obtained their informed consent. Participants had the right to know that their participation was voluntary and that they could withdraw from the study at any time. All studies in this paper did not involve live animals. The study did not involve human participants.

## Result analysis

In this paper, the simulation environment in gazebo is constructed based on the experimental environment of the real aircraft. During the training process, the position information (ENU system) and state information (yaw angle, altitude) of the UAV are randomly initialized at the beginning of each training act to ensure that the position of the target point in the UAV viewpoint is random in each act. This paper also provides an obstacle position initialization interface to verify that the UAV’s obstacle avoidance action is RL Agent makes local path planning from the lidar data to avoid away-from-boundary type obstacle avoidance effect.

The training body of this paper consists of two parts, the first part is used to verify that the UAV can realize the local path planning task with the help of the RL Agent in the case of such external environment information; that is, training is carried out without obstacles, lidar information and collision detection algorithms, and realizes the local path planning task; the second part adds obstacles in the environment and enables the radar data as well as the collision reward to realize a more complex and complete local path planning task. reward to realize a more complex and complete local path planning task. To eliminate the influence of model uncertainty on our experiments, we randomized the initial position of the UAV, the position of the target point, and the state and position of the obstacles in all our training and testing experiments. This approach minimizes the impact of model uncertainty on the experiments themselves. The above two parts of the experiment are equipped with an ordinary monocular camera below the UAV and a LiDAR above. The reward weights are fixed to $w_p = 20, w_E = 2, w_n = 20, w_C = -100$, and each output action interval is at [− 1,1], and the yaw angle interval is [$-180^{\circ }$,180$^{\circ }$] at initialization. The training algorithm is deployed in a computer equipped with an AMD Ryzen9 5900HX CPU, 32GB RAM, and GPU disabled, and the training environment is deployed into gazebo and uniformly controlled by HWT_OS commands.

### Simulation experiments in accessibility

In order to quantitatively evaluate the settings of the selected parameter configurations, this paper compares different environmental information, single-step reward noise values, and action noise values in an indoor environment. Under the premise that the training time is set to 8 h and the single-step reward noise value is set to 0.1, the training results of excluding the data preprocessing module are compared with those of adding the data preprocessing module; without limiting the training time, the training results of adding the data preprocessing module and setting the single-step reward noise value to 0.1 are compared with those of 0.5; without limiting the training time and adding the data preprocessing module, the single-step reward noise value is set to 0.5 and the initialized height threshold is set at [0.5]. to 0.5, and compare the training results with initialized height thresholds at [4m–6m] and [4m–12m]. Based on the training results, the average Q value, the maximum Q value and the convergence time are calculated.

**Figure 5:**
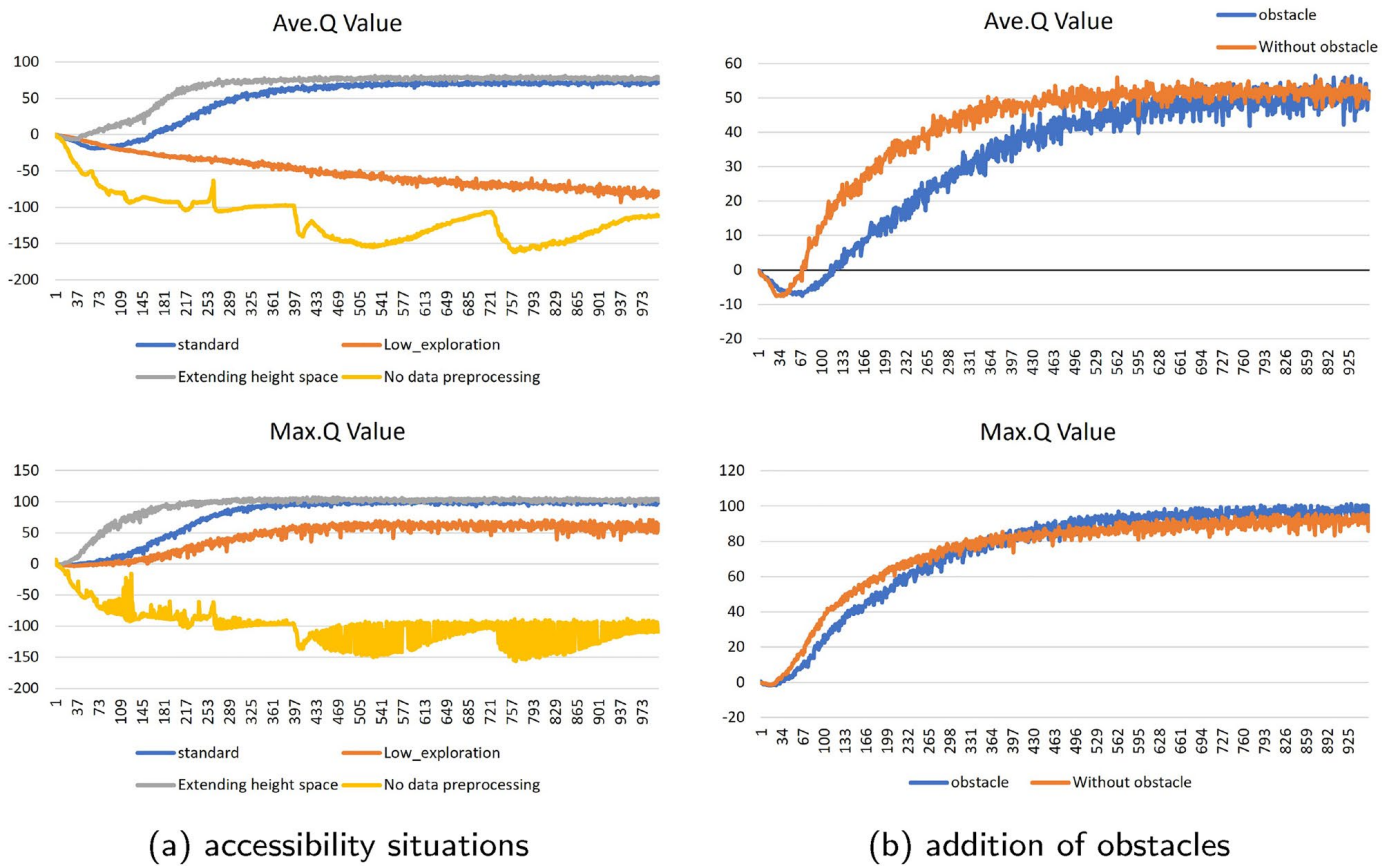
Max. Q and Ave.Q for simulation experiments.

As shown in Fig. 5a, when the state information preprocessing module is not intervened, the RL Agent is unable to make proper path planning actions, and the maximum Q value converges to a large negative value, while the intervention of the state information preprocessing module reduces the redundancy of the state information, so that there exists a chance for the RL Agent to reach the target point, but due to the pre-training period, the reward noise setting for it is too small, which inhibits its exploration process, so that the UAV exploration converges to the local optimum, and cannot make reasonable path planning actions in the edge zone; when the single-step reward noise value is adjusted higher, the convergence time of the maximum Q value is pushed back, but the average Q value converges in a higher interval, and the maximum Q value stabilizes at the maximum reward, so that the UAV can make suitable planning actions in the edge zone of the map to move towards the target point; when the altitude interval makes a change, the altitude when the height interval is changed, a bigger change in the interval means that the zoom ratio of the image becomes bigger, and the planning difficulty increases. As shown in Fig. 5, the average Q value and the maximum Q value also converge at 7h, and the convergence values are similar.

### Simulation experiments with obstacles

In order to verify the integrity of the algorithm local path planning, this paper adds 2 groups of obstacles in the indoor environment, and selects unrestricted training time, adding STATE information preprocessing module, single-step rewarding noise value set to 0.5, and initialization height threshold set to [4–6 m] as the baseline, and meanwhile, in order to increase the algorithm retrospective, the maximum number of steps in a single act is elevated to 10, and in the case of enabling lidar data, the environment augmented obstacle and unaugmented obstacle training results for comparison, according to the training results to calculate the average Q value, the maximum Q value.

As shown in Fig. 5b, this paper re-experiment after the subsequent incremental obstacles, Ave.Q Value of the beginning of the convergence time is significantly reduced, its planning action when encountering obstacles to make its points lower, but the final convergence effect is basically the same, in the value of 50 to achieve convergence. Q Value belongs to the decision made by the UAV in the last planning step, at this time, due to the close to the target point and away from the obstacles, so the convergence speed of Max.Q Value in the case of obstacles and no obstacles of the two do not have a big difference and ultimately converge to the maximum score.

As shown in Table 1, under the standard condition, i.e., no obstacles, the map side length is 5 m * 5 m, no limitation of training time, adding the data preprocessing module, setting the single-step reward noise value to 0.5, and the maximum number of exploring steps is 5, and the initial position of the UAV is randomized in each round; after 500 rounds of testing of the Agent, the UAV successfully achieves the success rate of path planning is 93%, which basically achieves the experiment of this paper. The purpose of this paper is to realize the autonomous local path planning task in unfamiliar environments, and in this map, only 1.73 steps are needed on average to achieve the path planning task; after adjusting the altitude interval, the same 500 rounds of Agent testing with random initial position of the UAV are also carried out, and the success rate and the successful average number of steps basically remain the same; this paper adopts the high-dimensional state space after isolating the data preprocessing module. space, the success rate is only 3%, which cannot realize the path planning task; when the single-step noise value is set to 0.1 to make it less exploratory, the success rate is only 13%, which is due to the fact that the UAV is trapped in a local optimum that it cannot get rid of during path planning.

**Figure 6:**
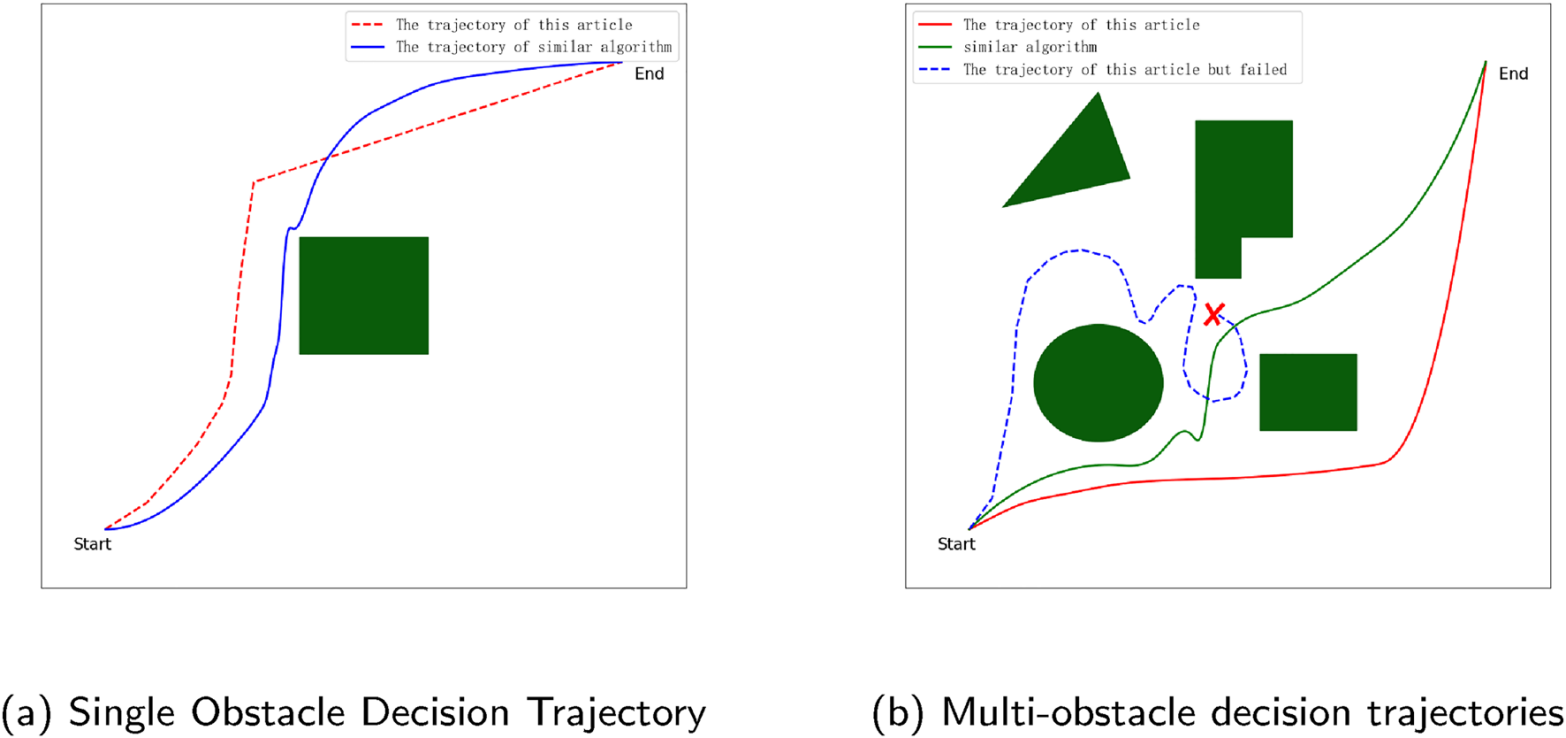
The trajectory plot of 2-dimensional space for UAVs.

**Table 1:** Planning success rate under each condition.

Condition	Success rate	Average steps
Standard	**93%**	**1.73**
With obstacle(average)	**92%**	**4.12**
With single obstacle	**93%**	**2.11**
With consecutive obstacle	**87%**	**8.31**
With dynamic obstacles	61%	1.26
Height interval extension	**90%**	**2**.**16**
High dimensional state space	3%	3.03
Low exploration rate	13%	3.50

Significant values are in bold.

#### With single obstacles and multiple consecutive obstacles

In this paper, this model is tested in single-obstacle and multiple-obstacle environments with random locations and random densities, respectively. As shown in Table 1, when a randomly located single obstacle is added to the environment, the UAV’s planning success rate is consistent with the performance of the no-obstacle environment, both achieving a 93% success rate. And from Fig. 6a, it can be seen that at this time the difference between the optimized paths of this paper and similar algorithms is not much, and both achieve good path planning work. And after deploying the model to the simulation environment with dense multiple obstacles added, as shown in Table 1, the success rate of planning can reach 87%, which is slightly lower compared to the single obstacle environment, but the number of planning steps is improved to 8.31. As can be seen from Fig. 6b, when the UAV plans a path to the middle of the multiple obstacles, there is a phenomenon that waits in place in order to minimize the penalty of the reward function, which is an important reason leading to the This is an important reason for the reduced success rate of UAV path planning. In contrast to similar algorithms, the algorithm in this paper tends to make the decision to move the UAV towards areas far away from obstacles before proceeding to reduce the risk of collision, while other algorithms tend to move the UAV away from the risk of collision without increasing the distance traveled by the UAV as much as possible.

#### With dynamic obstacles

Our model’s decision-making success rate was only 61% when deployed in a simulation environment with dynamic obstacles, as shown in Table 1. Our analysis indicates that there are two main factors responsible for the significant reduction in planning success rate in the dynamic obstacle environment.

Firstly, the single-step planning approach of the algorithm, which treats each planning step as relatively independent, makes it impossible to accurately predict the trajectory of dynamic obstacles. Secondly, the real-time planning nature of the algorithm is not sufficient to continuously predict the dynamic obstacles and achieve the desired level of decision-making. These are the two primary reasons why the model fails to produce good results in dynamic obstacle environments.

### Experimental results

Based on the experiments conducted, this paper’s algorithm has shown impressive performance in scenarios without obstacles, a static single obstacle, and multiple continuous obstacles. Compared to other UAV path planning algorithms, this algorithm has greater portability as it supports the HWT_OS system. This means that the model can be directly deployed to other UAVs without changing the UAV’s native controller. Additionally, this algorithm leverages the RL Agent, which enables the UAV to make autonomous decisions at a higher level and is comparable to traditional path planning algorithms. Furthermore, it tends to move the UAV away from all obstacles before moving towards the target direction. However, the algorithm in this paper has limitations when it comes to dynamic obstacle environments. The reason is that the algorithm mainly uses the Markov decision process for discontinuous decision-making, which limits the continuous decision-making speed of the UAV when facing dynamic obstacles. Consequently, this algorithm’s planning effectiveness is poor in such scenarios.

## Conclusion and future work

This paper presents an autonomous local path planning algorithm for UAVs, based on the TD3 algorithm. The algorithm can help UAVs quickly and accurately plan their path in unfamiliar environments. Additionally, this algorithm is highly portable, thanks to the HWT_OS system proposed in this paper. It can work in other UAV devices by modifying only some of the parameters, without changing the native controller of the UAV.

And we will further improve the decision speed of the reinforcement learning agent in the future to improve decision-making in dynamic obstacle environments and expand it to set UAV planning scenarios (Supplementary Informations S1 and S2).

### Supplementary Information

Supplementary Information 1.

Supplementary Information 2.

## Data Availability

All data generated or analysed during this study are included in this published article and its supplementary information files.
